# Proteomic profile of follicular fluid from patients with polycystic ovary syndrome (PCOS) submitted to *in vitro* fertilization (IVF) compared to oocyte donors

**DOI:** 10.5935/1518-0557.20190041

**Published:** 2019

**Authors:** Thais S Domingues, Tatiana CS Bonetti, Daniel C Pimenta, Douglas O C Mariano, Bruna Barros, Ana Paula Aquino, Eduardo L A Motta

**Affiliations:** 1 Departamento de Ginecologia. Universidade Federal de São Paulo - Escola Paulista de Medicina (UNIFESP-EPM). São Paulo - SP, Brazil; 2 Huntington Medicina Reprodutiva. São Paulo - SP, Brazil; 3 Laboratório de Bioquímica e Biofísica, Instituto Butantan. São Paulo - SP, Brazil

**Keywords:** polycystic ovary syndrome, proteomic, follicular fluid, *in vitro* fertilization

## Abstract

**Objective:**

The follicular fluid (FF) of women with polycystic ovary syndrome (PCOS) seems to exhibit a profile different from that of fertile women, which may be related to folliculogenesis disruption in PCOS patients. The aim of this study was to evaluate the differentially expressed proteins in the FF of women with PCOS compared to oocyte donors (ODs).

**Methods:**

This screening study included thirteen (13) women who underwent *in vitro* fertilization (IVF) cycles: seven (7) ODs and six (6) PCOS patients. The patients underwent standard ovarian stimulation, and the FF was analysed using ion trap and time-of-flight liquid chromatography-mass spectrometry (LCMS-IT-TOF).

**Results:**

The FF of the patients was matched to 229 proteins, with 61 proteins exclusive to the PCOS group, 123 proteins exclusive to the ODs, and 45 proteins found in both groups. We highlight fetuin-A and vitamin D ligand protein, which were exclusively expressed in the PCOS group; Complement C3 overexpressed in the PCOS group; and 26S protease only expressed in the OD group. The canonical pathways LXR/RXR activation, FXR/RXR activation, prothrombin activation are directly related to the disrupted metabolism and increased inflammatory status found in PCOS patients.

**Conclusions:**

The findings of the differentially expressed proteins and matched pathways are associated with folliculogenesis, indicating it relevance to oocyte quality.

## INTRODUCTION

Polycystic ovary syndrome (PCOS) is characterized by hyperandrogenism, ovulation disorder and polycystic ovaries (PCO) and the exclusion of other endocrinopaties (Rotterdam ESHRE/ASRM-Sponsored PCOS Consensus Workshop Group, 2004). PCOS affects 6-8% of women of reproductive age. Although PCOS was first described eighty years ago (Stein & Leventhal, 1935), its aetiology is not yet fully elucidated, as it is a heterogeneous and complex disorder with metabolic and reproductive implications. PCOS represents the major ovulatory cause of infertility, which leads some PCOS patients to pursue *in vitro* fertilization (IVF) treatments (Dumesic *et al*., 2015).

The follicular fluid (FF) that surrounds the cumulus-oocyte complex contains several factors that originate from the blood transudate and are secreted by cumulus cells, such as proteins, steroids, polysaccharides and other metabolites; thus, FF provides a unique microenvironment in which to study oocyte development and maturation (Schweigert *et al.*, 2006; Appasamy *et al.*, 2008)

It is recognized that the FF from women with PCOS is characterized by deregulated expression of several compounds, including anti-Müllerian hormone (AMH), inhibin-B, activin-A, amphiregulin, heparan sulfate proteoglycan 2; tumour necrosis factor (TNF), α-induced protein 6 and plasminogen (Ambekar *et al*., 2015). Although previous studies have identified molecules in the FF of PCOS patients that are associated with the deregulation of follicle maturation, this process is not completely understood. We aimed to identify putative differences in the FF profiles of PCOS patients and fertile women, represented by egg donors, using mass spectrometric analysis to better understand the mechanisms that lead to deregulated oocyte development.

## MATERIAL AND METHODS

### Study design

This prospective study evaluated the protein components of FF from oocyte donors (ODs) in comparison to those of FF from infertile women with PCOS who underwent IVF at Huntington Reproductive Medicine Centre and the Reproductive Unit of the Federal University of São Paulo (UNIFESP) from 2012 to 2015. This study protocol was approved by the ethics committee of Federal University of São Paulo (No. 1620/2011), and informed written consent was obtained from each patient.

### Casuistic

Thirteen (13) patients were enrolled and divided into two groups: ODs (n=7) and infertile PCOS patients (PCOS; n=6). The ODs were healthy female volunteers under the age of 32 years with body mass indices between 18 and 30 kg/m^2^, antral follicle counts ≥10, normal karyotypes, and the absence of endometriosis who had been screened and tested for infectious diseases. The PCOS patients were diagnosed with infertility according the Rotterdam criteria (Rotterdam ESHRE/ASRM-Sponsored PCOS Consensus Workshop Group, 2004). All the PCOS patients presented body mass indices (BMI) below 25 kg/m^2^, basal follicular stimulating hormone (FSH) levels below 15 IU/L, basal oestradiol levels below 50 pg/mL, the presence of both ovaries, and no ongoing infectious diseases or uterine abnormalities, and they had undergone intracytoplasmic sperm injection (ICSI) cycles with ejaculated sperm. For both groups, patients who presented gynaecological bleeding, hydrosalpinx, allergy to gonadotropins or other medications used in the treatment, severe oligo- or azoospermia, abusive use of any medications or ovarian hyperstimulation syndrome (OHSS) during the treatment were excluded.

### Ovarian stimulation protocol and sample collection

FF was obtained from women who underwent the standard short protocol of IVF (using a GnRH antagonist - Cetrotide^®^, Merck, Germany). Controlled ovarian stimulation was performed using recombinant FSH (rFSH - Gonal-F^®^, Merck, Germany) and was monitored with ultrasound. Ovulation was triggered with a GnRH agonist (aGnRH - Gonapeptyl, Ferring, Germany) when at least two follicles reached 20 mm. The FF was collected from the dominant follicles through aspiration between 34 and 36 h after aGnRH administration, using transvaginal ultrasound guidance. Only clear FF samples, without blood or flushing medium contamination, were processed. The selected FF samples were centrifuged at 1200 rpm for 10 to 15 min to remove cellular debris. The supernatants were stored at −80ºC until purification.

### Protein extraction

Before analysis, albumin and immunoglobulins were removed from the FF samples (25 µL) using the Albumin & IgG Depletion SpinTrap (GE Healthcare Life Sciences™) according to the manufacturer’s protocol. The protein concentration in each FF sample was measured in triplicate using a bicinchoninic acid assay (BCA assay) (Smith *et al*., 1985). Twenty-five to thirty micrograms of albumin/IgG-depleted FF protein was subjected to electrophoresis via 12.5% sodium dodecyl sulfate-polyacrylamide gel electrophoresis (SDS-PAGE) under reducing conditions (Laemmli, 1970) and stained with Coomassie brilliant blue R-250.

 All gels were analysed, and ten bands were cut equally for each sample and processed separately for in-gel digestion according to the protocol described by Westermeier *et al*. (2002), with slight modifications.

### Mass spectrometry analysis

Liquid chromatography-mass spectrometry (LC-MS) analyses were performed using an Electrospray-Ion Trap-Time of Flight system (ESI-IT-TOF) (Shimadzu Co., Japan) equipped with a binary Ultra-Fast Liquid Chromatography system (UFLC) (20A Prominence, Shimadzu) at the Laboratory of Biochemistry and Biophysics of the Butantan Institute (São Paulo, Brazil). First, each band sample was lyophilized, resuspended in 50 µL of 0.1% acetic acid and loaded on a C18 column (Discovery C18, 5 µm; 50 × 2.1 mm) in a binary solvent system: (A2) water/acetic acid (999/1, v/v) and (B2) ACN/water/acetic acid (900/99/1, v/v/v). The column was eluted at a constant flow rate of 0.2 mL.min−1 with a 5 to 70% gradient of solvent B2 over 35 min. The eluates were monitored by a Shimadzu SPD-M20A PDA detector before introduction into the mass spectrometer. The interface voltage was adjusted to 4.5 kV, and the capillary voltage was 1.76 kV at 200ºC. MS spectra were acquired in positive mode and collected in the 80-2000 mass charge (m/z) range. MS/MS spectra were collected in the 50-1950 m/z range. Instrument control, data acquisition, and data processing were performed with LabSolutions (LCMSsolution 3.60.361 version, Shimadzu).

### Bioinformatics analysis

Proteomic analysis was performed using the Mascot Server (ion search) in house version (2.4) and Peaks Studio V7 (Bioinformatics Solutions, Inc., Waterloo, Canada). The following parameters were adjusted for the search: parent mass and fragment mass error tolerance: 0.1 Da; enzyme: trypsin; fixed modification: carbamidomethylation; variable modification: methionine oxidation; precursor mass search type: monoisotopic; max missed cleavages: 3; non-specific cleavages: one; database: SwissProt, taxon: *Homo sapiens*; peptide - 10 lgP: ≥15; and protein - 10 lgP: ≥20. The false discovery rate (FDR) for peptide-spectrum matches was ≤1%.

Although each band was analysed separately in LC-MS, we performed a protein search combining all ten bands obtained from each patient. A protein was considered exclusive when it was detected in the FF of patients in either the OD or PCOS group and was totally absent in all of the samples from the other group; a protein was considered overexpressed when it was detected in both groups, but one group had a mean detected peptide level greater than that of the other group by 50% (greater than 1.5-fold in one group and less than 0.5-fold in the other group).

### Protein identification and classification

The identified proteins were classified according to their classes, locations, biological functions and processes using the PANTHER Classification System (Gene Ontology Phylogenetic Annotation Project, Los Angeles, USA) (Mi *et al*., 2016). System biology analysis was carried out using Ingenuity™ Pathway Analysis software (IPA™, QIAGEN, Redwood, USA). The overexpressed proteins were selected for the analysis of canonical pathways and biological interaction networks. The biological processes were staggered according to the IPA™ Knowledge Base. The associations between the identified proteins and canonical pathways in the database were assessed with Ingenuity™ software using Fisher's exact test (significance of *p*<0.01).

### Statistical analysis

Clinical proteomic studies is a multistage biomarker pipeline that begin with the identification of a large number of proteins in a small set of sample. This screening step, as this is our study, the number of samples included was based on the principle that a minimum number of samples considering biological and technical variation inherent in the experiment. Thus, we included a small number of samples and non-parametric statistic was applied.

The patients’ demographic data were evaluated using descriptive statistics. Normality was evaluated with the Kolmogorov-Smirnov test. Non-paired continuous data were compared using the Mann-Whitney test for means comparisons and paired data were compared using Wilcoxon’s signed-rank test. Data analyses were performed using SPSS 22 (IBM SPSS Software, USA), and significance was accepted for *p*-values ≤0.05.

## RESULTS

The patients’ demographics and clinical outcomes are described in Table 1. The ovarian reserves of the patients in both groups had similar profiles in terms of basal FSH dosages and antral follicle counts. As expected, the OD patients were younger, and the PCOS patients had longer menstrual cycle intervals. The parameters related to ovarian induction (length, serum hormone levels, and mature (metaphase II-MII) oocytes collected) were similar between the groups, except for the amount of gonadotropin administered, which was higher in the OD group. The OD group had a higher number of top-quality embryos (3^rd^ day) than the PCOS group.

**Table 1:** Demographic and clinical data for the patients in the PCOS and OD groups

	OD group	PCOS group	*p*-value
Number of samples	7	6	----
Age (years)	24.71±3.45	33.50±1.38	0.0050
BMI (kg/m^2^)	24.03±1.15	21.37±2.02	0.0264
Menstrual cycle interval (days)	29.29±1.60	50.00±21.68	0.0033
Antral follicle count	14.0±4.6	14.8±4.8	0.7723
Basal FSH (UI/mL)	5.56±1.41	5.97±1.44	0.5180
Gonadotropin dose (UI)	2829.0± 494.9	1758.0±1097.0	0.0513
Oocyte induction length (days)	12.0±0.0	11.7±1.9	0.4282
Oestradiol (E2)* (nmol/L)	3503±1777	2892±2474	0.7308
Progesterone (P4)* (nmol/L)	2.74±1.40	2.24±1.278	0.4295
MII oocytes collected	10.86±2.27	13.33±3.39	0.1277
Top-quality embryos (D3)	3.4±14.8	2.2±2.4	0.0343

* Measured prior to oocyte collection.

The proteomic analysis of the proteins from the FF samples matched 229 proteins in the SwissProt database. Forty-five (45) proteins were detected in both groups. Three of these shared proteins were excluded from analyses, as they were contaminants (trypsins and keratins), resulting in 42 proteins shared between the two groups. There were 61 proteins that were exclusive to the PCOS group, and 123 proteins that were exclusive to the OD group (Supplemental Tables I and II). To refine the SwissProt results, only proteins that were expressed in at least two patients from each group were considered. Five proteins were selected from those exclusively expressed in the PCOS group, and three proteins were selected from those exclusively expressed in the OD group (Table 2).

**Table 2:** Proteins that were exclusively detected in at least two FF samples from either the PCOS or OD group

Group	SwissProt ID	Description	Peptide mean	Average mass (Da)	Patients
**PCOS**	Q17R60	Interphotoreceptor matrix proteoglycan 1	1.5	89387	2
	Q8 WXI7	Mucin-16	1.3	2.00E_+06_	3
	P02765	Alpha-2-HS glycoprotein	1.5	39325	2
	P02774	Vitamin D-binding protein	2.0	52964	2
	Q15020	S Squamous cell carcinoma antigen recognized by T-cells 3	1.0	109935	2
**OD**	P43686	26S protease regulatory subunit 6B	1.5	47366	2
	Q8IWI9	MAX gene-associated protein	1.0	331836	2
	Q8IWI9	Transformation/transcription domain - associated protein	1.0	437603	2

The differentially expressed proteins were rated and selected. Six proteins were the highest occurring peptides in the PCOS group, and ten proteins were the highest occurring peptides in the OD group (Table 3). The most significant proteins, which were expressed in the FF of at least two patients, were the complement C3 protein, which was overexpressed in the PCOS group, and titin, serum albumin, complement C4-A, complement C4-B, alpha-1-acid glycoprotein 1 and alpha-2-macroglobulin, which were overexpressed in the OD group.

**Table 3:** Proteins that were differentially expressed in the FF of PCOS and OD patients and for which one group had at least 50% more peptides than the other

SwissProt ID	Description	Peptide mean		Fold change
		OD	PCOS	
P04004	Vitronectin	1	4	4.0
P25311	Zinc-alpha-2 glycoprotein	1	2	2.0
P98160	Basement membrane-specific heparan sulfate proteoglycan core protein	1	2	2.0
Q6 V0I7	Protocadherin Fat 4	1	2	2.0
P01834	Complement factor B	4	7	1.8
P01024	Complement C3	5.3	8	1.5
Q8 WZ42	Titin	3	2	0.6
Q9C0G6	Dynein heavy chain 6, axonemal	3	2	0.6
P02768	Serum albumin	16.75	10	0.6
P0C0 L4	Complement C4-A	4	2	0.5
P0C0 L5	Complement C4-B	4	2	0.5
P02763	Alpha-1-acid glycoprotein 1	2.5	1	0.4
P01023	Alpha-2-macroglobulin	5.75	1	0.2

The proteins that were identified as exclusive or overexpressed were classified according to the Gene Ontology database and analysed with respect to biological pathways with the Ingenuity™ software. Six molecular functions were identified, and four of them were very similar between the two groups (GO:0005488, GO:0004872, GO:0005198 and GO:0003824). The PCOS patients had fewer proteins related to transporter activity (GO:0005215) (7.10% OD *vs.* 2.30% PCOS). Additionally, translation regulation activity (GO:0045182) was detected only in the OD patients (1.80%) but was represented by only one protein.

**Figure 1:**
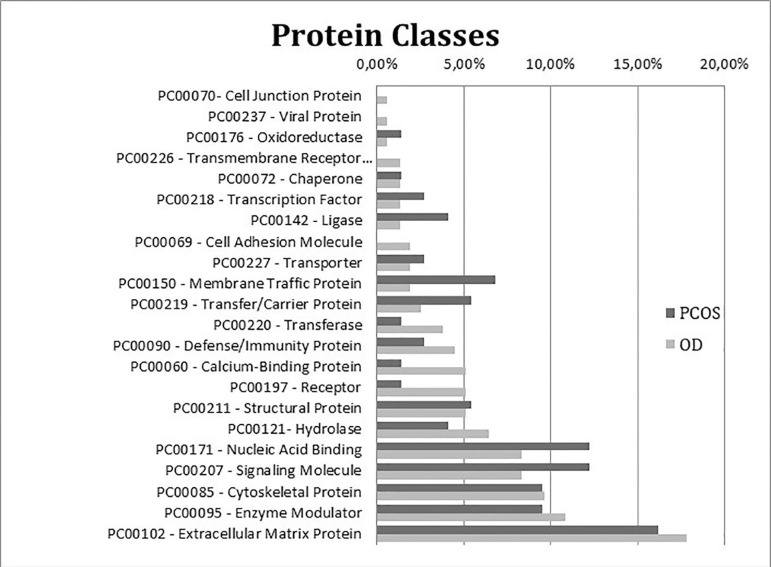
Chart indicating the percentages of exclusive and upregulated FF proteins from the PCOS (dark grey) and OD (light grey) groups classified according to protein classes based on the Gene Ontology database

**Figure 2:**
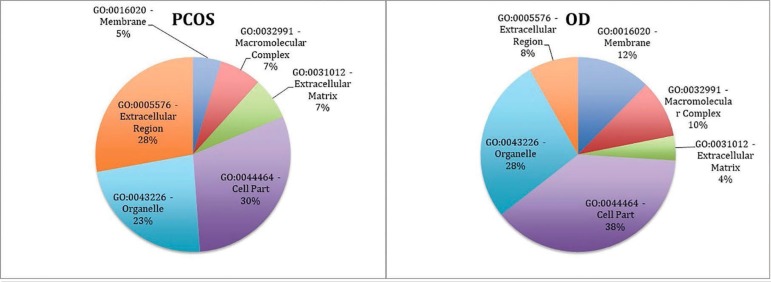
Chart indicating the percentages of exclusive and upregulated FF proteins from the PCOS and OD groups, classified according to cellular components based on the Gene Ontology database

The evaluation of protein classes resulted in nineteen different classes (Figure 1). The most representative classes for the OD group were cell junction, cell adhesion and transmembrane receptor regulatory/adaptor, which were exclusive to this group. The PCOS group presented more proteins related to the oxireductase, membrane traffic protein and ligase classes. The distribution of the protein classes in terms of cellular components differed between the groups: the PCOS group had more extracellular proteins, and the OD group had more membrane and membrane-related proteins (Figure 2).

**Figure 3:**
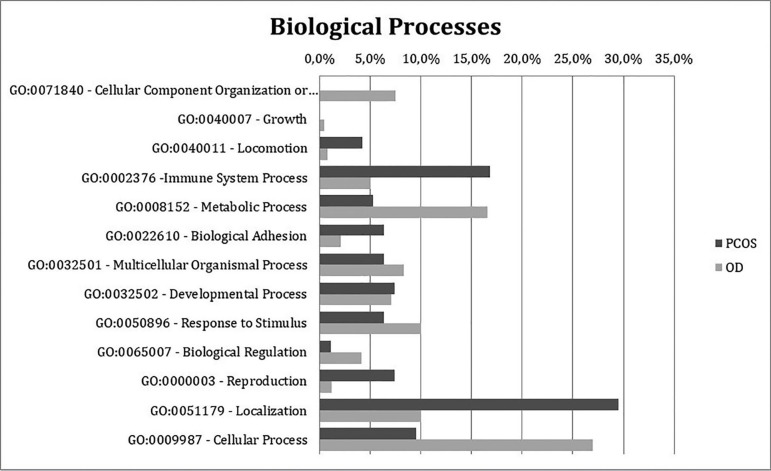
Chart indicating the percentages of exclusive and upregulated FF proteins from the PCOS (dark grey) and OD (light grey) groups classified according to biological processes based on the Gene Ontology database

The biological processes associated with the detected proteins differed remarkably between the groups (Figure 3). The PCOS group had more proteins associated with immune process, cell localization and biological adhesion molecules. The OD group had more proteins associated with metabolic processes and cell component organization, suggesting that the OD group was more metabolically active.

These results were corroborated by the biological pathway analysis (Table 4), as the proteins identified in the FF of the OD patients were related to cellular assembly and organization and cellular function and maintenance. The PCOS group had fewer proteins matched to cellular assembly and organization. As expected, the proteins of the OD group matched biological functions related to embryo and general organism development; only two of these proteins were detected in the FF of the patients in the PCOS group. The main canonical pathways (Supplemental Table III) found only for the proteins in the FF from the PCOS patients were LXR/RXR activation (*p*=9.04 E^-11^, overlap 7%) and FXR/RXR activation (*p*=1.67 E^-10^, overlap 6.6%), which are key for the metabolism of lipids, lipoproteins and glucose, reflecting the disrupted metabolism exhibited by PCOS patients. In addition, proteins associated with the intrinsic (*p*=1.70E^-07^, overlap 22.2%) and extrinsic (*p*=1.48E^-06^, overlap 13.3%) prothrombin activation pathways were identified in the FF from the PCOS group.

**Table 4:** Molecular and cellular function and physiological system development and function matched to the proteins detected in the FF from PCOS and OD patients

	PCOS		OD	
	*p*-value	Molecules	*p*-value	Molecules
**Molecular and Cellular Function**				
Carbohydrate Metabolism	8.43E^-03^ – 1.94E^-06^	10		
Lipid Metabolism	8.43E^-03^ – 1.94E^-06^	11		
Small Molecule Biochemistry	8.43E^-03^ – 1.94E^-06^	11		
Cell-To-Cell Signalling and Interaction	8.43E^-03^ – 3.52E^-06^	15		
Cellular Assembly and Organization	8.43E^-03^ – 8.38E^-06^	24	1.19E^-02^ – 3.89E^-05^	45
Cellular Function and Maintenance			1.14E^-02^ – 3.89E^-05^	38
Post-Translational Modification			8.95E^-03^ – 9.71E^-05^	3
Protein Degradation			9.71E^-05^ – 9.71E^-05^	2
Protein Synthesis			8.95E^-03^ – 9.71E^-05^	2
**Physiological System Development and Function**				
Embryonic Development	8.43E^-03^ – 3.52E^-06^	14	1.14E^-02^ – 1.94E^-04^	29
Haematological System Development and Function	8.43E^-03^ – 6.08E^-06^	18		
Hair and Skin Development and Function	5.63E^-03^ – 7.13E^-06^	9		
Tissue Development	8.43E^-03^ - 8.38E^-06^	21	1.24E^-02^ – 1.94E^-04^	33
Renal and Urological System Development and Function	5.63E^-03^ – 1.19E^-05^	10		
Nervous System Development and Function			1.14E^-02^ – 1.94E^-04^	22
Organ Development			1.14E^-02^ – 1.94E^-04^	20
Organismal Development			11.14E^-02^ – 1.94E^-04^	32

## DISCUSSION

 Our findings showed significantly diminished expression of proteins involved in key processes associated with oocyte competence and embryo development in PCOS patients. In addition, overexpression of proteins related to oxidative stress, the immune response and lipid, lipoprotein and carbohydrate metabolism was observed in these patients. Although many proteomics analyses of FF have been published recently, the functional correlations among these proteins are still poorly recognized. We attempted to correlate the differentially expressed proteins in the FF from PCOS patients with physiological pathways. We believe that the observed differences may reflect the PCOS patients’ diminished embryo quality, as this factor is directly reliant on oocyte characteristics.

 In our study, the inflammatory pathway represented by complement C3 protein and vitronectin was overexpressed in the FF from the PCOS group. The augmented levels of these proteins in the FF seems to be related to poor oocyte quality, potentially explaining IVF failure (Estes *et al*., 2009). Additionally, excess complement cascade activation leads to deficiencies in vascular endothelial growth factor (VEGF) activity, which is essential for proper oocyte maturation (Jarkovska *et al*., 2010). Another marker for the disruption of the inflammatory pathway in PCOS patients is the overexpression of alpha-2-HS-glycoprotein (fetuin-A). This protein is an acute-phase inflammatory regulator that is usually upregulated in OHSS (Jarkovska *et al*., 2011). As we excluded OHSS patients and applied a GnRH agonist analogue to trigger ovulation, the presence of fetuin-A was not expected and may contribute to the decreased oocyte quality in those patients.

Moreover, the poor oocyte quality and deregulated inflammatory status of PCOS patients may be related to the overexpression of vitamin D-binding protein (VDBP) in their FF. VDBP was another protein found exclusively in the FF of the PCOS group, and according to the literature, this protein may be related to decreased implantation, pregnancy (Estes *et al*., 2009), and live birth rates (Benkhalifa *et al*., 2015); VDBP is even more strongly associated with a higher risk of miscarriages (Kushnir *et al*., 2012) and foetal growth restriction (Wookey *et al*., 2017).

The overexpressed coagulation pathway found in PCOS-FF, characterized by intrinsic and extrinsic prothrombin activation, is also linked to an inflammatory response; this pathway has important roles in follicle physiology (de Agostini, 2006) and may be associated with poor IVF outcomes (Bianchi *et al*., 2016).

The exclusive and overexpressed proteins in the OD group, such as 26S protease, alpha-1-acid glycoprotein 1 and alpha-2-macroglobulin, are correlated with a better ovarian stimulation response. The 26S protease is a highly specialized, conserved ribonucleoprotein that facilitates assembly of proteasome complexes; this protein is directly and indirectly involved in the regulation of gene expression (Mittenberg, 2014). Alpha-2-macroglobulin is linked to intrinsic and extrinsic coagulation cascades and is correlated with the complement pathway (Hanrieder *et al.*, 2009). The adequate regulation of coagulation and immune response pathways is essential for the extracellular matrix (ECM) modelling that facilitates follicular growth, ovulation and corpus luteum formation (Kamat *et al*., 1995; Curry & Smith, 2006), which may be more effective in fertile women.

Furthermore, PCOS is frequently associated with disrupted lipid and carbohydrate metabolism (Dumesic *et al*., 2015). We found some proteins in the PCOS-FF that were absent in the OD group; these proteins represented metabolic pathways, and their presence corroborated previous findings (Dai & Lu, 2012; Ambekar *et al*., 2015). Our findings at the FF level suggest that the impairment of lipid and lipoprotein metabolism also occurs within a specific microenvironment, such as that of infertile women with PCOS and a normal BMI. The increased inflammatory status and metabolic disruption observed through the protein composition of the FF from our PCOS patients seem to lead to a worse prognosis for oocyte viability and may affect IVF outcomes. Previous studies of PCOS patients undergoing IVF treatment obtained a great number of oocytes but reported poor fertilization and embryo development rates, an outcome that may be linked to deregulated oocyte activation through a damaged microenvironment (Jungheim *et al*., 2009).

To find potential markers of oocyte quality, our inclusion and exclusion criteria were very strict to allow us to identify markers that are exclusive to PCOS-FF without overlapping with other pathologies and conditions, such as obesity or OHSS. We hypothesize that the evaluation of fetuin-A, VDBP, complement C3 and 26S protease expression in the FF of PCOS patients undergoing IVF could be associated with oocyte quality. The limitations of these findings include the absence of experimental validation of the candidate markers through other techniques, such as Western blotting; additionally, the differentially expressed proteins must be correlated with the final IVF outcomes to endorse their use in clinical practice.

## Appendix

**Supplementary Table I:** List of proteins identified exclusively in the FF from PCOS group in comparison to OD group.

SwissProt ID	Name	Class	Molecular Function	Cellular compartment	Number of Patients
Q17R60	Interphotoreceptor matrix proteoglycan 1	Glicoproteína de matriz extracelular (PC00102); receptor (PC00100)	receptor activity(GO:0004872)	extracellular region(GO:0005576)	3
Q8WXI7	Mucin-16	NA	NA	NA	3
PNA1NA42	Complement C3	NA	cysteine-type endopeptidase inhibitor activity(GO:0003824)	#N/D	2
P02765	Alpha-2-HS-glycoprotein	Inibidor de cisteína protease (PC00095); Glicoproteína de matriz extracelular (PC00191)	peptidase inhibitor activity(GO:0003824)	extracellular space(GO:0005576)	2
P02774	Vitamin D-binding protein	NA	NA	NA	2
Q15020	Squamous cell carcinoma antigen recognized by T-cells 3	NA	NA	NA	2
A6NDX4	Putative transmembrane protein	NA	NA	#N/D	1
O14526	FCH domain only protein 1	Proteína de citoesqueleto da família das actinas (PC00085); proteína reguladora de *trafficking* de membrana (PC00041)	NA	NA	1
O14578	Citron Rho-interacting kinase	non-receptor serine/threonine protein kinase(PC00220)	protein kinase activity(GO:0003824)	NA	1
O15020	Spectrin beta chain, non-erythrocytic 2	non-motor actin binding protein(PC00085)	actin binding(GO:0005488);structural constituent of cytoskeleton(GO:0005515)	intracellular(GO:0044464)	1
O15357	Phosphatidylinositol 3,4,5-trisphosphate 5-phosphatase 2	phosphatase(PC00121)	NA	NA	1
O75445	Usherin	extracellular matrix linker protein(PC00102);receptor(PC00101)	receptor activity(GO:0004872)	extracellular matrix(GO:0031012);extracellular region(GO:0005576)	1
O75691	Small subunit processome component 20 homolog	NA	NA	intracellular(GO:0044464);nucleolus(GO:0005622);ribonucleoprotein complex(GO:0043226)	1
P00739	Haptoglobin-related protein	annexin(PC00060);calmodulin(PC00050);peptide hormone(PC00131);protease inhibitor(PC00061);receptor(PC00207);serine protease(PC00179)	NA	NA	1
P01008	Antithrombin-III	serine protease inhibitor(PC00095)	serine-type endopeptidase inhibitor activity(GO:0003824);serine-type peptidase activity(GO:0016787)	extracellular space(GO:0005576)	1
P01019	Angiotensinogen	serine protease inhibitor(PC00095)	serine-type endopeptidase inhibitor activity(GO:0003824);serine-type peptidase activity(GO:0016787)	extracellular space(GO:0005576)	1
P02538	Keratin, type II cytoskeletal 6A	intermediate filament(PC00085);structural protein(PC00129)	structural constituent of cytoskeleton(GO:0005198)	intermediate filament cytoskeleton(GO:0043226);intracellular(GO:0005856)	1
P02671	Fibrinogen alpha chain	signaling molecule(PC00207)	receptor binding(GO:0005488)	extracellular region(GO:0005576)	1
P02675	Fibrinogen beta chain	signaling molecule(PC00207)	receptor binding(GO:0005488)	extracellular region(GO:0005576)	1
P02679	Fibrinogen gamma chain	NA	NA	NA	1
P02790	Hemopexin	transfer/carrier protein(PC00219)	NA	extracellular matrix(GO:0031012)	1
P04259	Keratin, type II cytoskeletal 6B	intermediate filament(PC00085);structural protein(PC00129)	structural constituent of cytoskeleton(GO:0005198)	intermediate filament cytoskeleton(GO:0043226);intracellular(GO:0005856)	1
P08574	Cytochrome c1, heme protein, mitochondrial	NA	NA	cytoplasm(GO:0044464);mitochondrion(GO:0005622)	
P08603	Complement factor H	NA	NA	NA	1
P09529	Inhibin beta B chain	growth factor(PC00207)	transforming growth factor beta receptor binding(GO:0005488)	extracellular space(GO:0005576)	1
P13645	Keratin, type I cytoskeletal 10	intermediate filament(PC00085);structural protein(PC00129)	structural constituent of cytoskeleton(GO:0005198)	intermediate filament cytoskeleton(GO:0043226);intracellular(GO:0005856)	1
P19827	Inter-alpha-trypsin inhibitor heavy chain H1	serine protease inhibitor(PC00095)	protein binding(GO:0005488);serine-type endopeptidase inhibitor activity(GO:0005515)	NA	1
P20742	Pregnancy zone protein	complement component(PC00090);cytokine(PC00078);serine protease inhibitor(PC00207)	cytokine activity(GO:0005488);serine-type endopeptidase inhibitor activity(GO:0005515)	NA	1
P27169	Serum paraoxonase/arylesterase 1	NA	hydrolase activity, acting on ester bonds(GO:0003824)	NA	1
P33991	DNA replication licensing factor MCM4	DNA helicase(PC00171);hydrolase(PC00009)	DNA helicase activity(GO:0003824);hydrolase activity(GO:0004386);nucleic acid binding(GO:0003678)	NA	1
P35908	Keratin, type II cytoskeletal 2 epidermal	intermediate filament(PC00085);structural protein(PC00129)	structural constituent of cytoskeleton(GO:0005198)	intermediate filament cytoskeleton(GO:0043226);intracellular(GO:0005856)	1
P48668	Keratin, type II cytoskeletal 6C	intermediate filament(PC00085);structural protein(PC00129)	structural constituent of cytoskeleton(GO:0005198)	intermediate filament cytoskeleton(GO:0043226);intracellular(GO:0005856)	1
P49454	Centromere protein F	NA	NA	NA	1
P49589	Cysteine--tRNA ligase, cytoplasmic	RNA binding protein(PC00171);aminoacyl-tRNA synthetase(PC00031)	aminoacyl-tRNA ligase activity(GO:0003824)	cytosol(GO:0044464)	1
P49842	Serine/threonine-protein kinase 19	NA	NA	NA	1
P78312	Protein FAM193A	NA	NA	NA	1
P98170	E3 ubiquitin-protein ligase XIAP	protease inhibitor(PC00095)	cysteine-type endopeptidase inhibitor activity(GO:0003824);ubiquitin-protein ligase activity(GO:0016787)	cytoplasm(GO:0044464);microtubule(GO:0005622);nucleus(GO:0005737)	1
Q12873	Chromodomain-helicase-DNA-binding protein 3	DNA helicase(PC00171)	NA	NA	1
Q13129	Zinc finger protein Rlf	nuclease(PC00171);transcription factor(PC00170)	nuclease activity(GO:0003824);sequence-specific DNA binding transcription factor activity(GO:0016787)	NA	1
Q15813	Tubulin-specific chaperone E	chaperone(PC00072)	NA	NA	1
Q3KP22	Uncharacterized protein C11orf85	NA	NA	NA	1
Q53GS7	Nucleoporin GLE1	nucleic acid binding(PC00171);transfer/carrier protein(PC00219)	nucleic acid binding(GO:0005488)	NA	1
Q63HN8	E3 ubiquitin-protein ligase RNF213	NA	NA	NA	1
Q6YP21	Kynurenine--oxoglutarate transaminase 3	transaminase(PC00220)	transaminase activity(GO:0003824)	NA	1
Q70CQ2	Ubiquitin carboxyl-terminal hydrolase 34	NA	NA	NA	1
Q7Z6M1	Rab9 effector protein with kelch motifs	NA	NA	NA	1
Q86YQ8	Copine-8	membrane traffic protein(PC00150)	NA	NA	1
Q8IWT3	Cullin-9	NA	NA	NA	1
Q8IY50	Putative thiamine transporter SLC35F3	NA	NA	NA	1
Q8N9W7	Putative transmembrane protein FLJ36131	NA	#N/D	#N/D	1
Q8NHM4	Putative trypsin-6	serine protease(PC00121)	serine-type peptidase activity(GO:0003824)	extracellular space(GO:0005576)	1
Q8TDI0	Chromodomain-helicase-DNA-binding protein 5	DNA helicase(PC00171)	NA	NA	1
Q8TF01	Arginine/serine-rich protein PNISR	NA	NA	NA	1
Q92665	28S ribosomal protein S31, mitochondrial	NA	NA	NA	1
Q9H1H9	Kinesin-like protein KIF13A	microtubule binding motor protein(PC00085)	microtubule motor activity(GO:0003824)	cytoskeleton(GO:0043226);intracellular(GO:0005856);protein complex(GO:0044464)	1
Q9NQ66	1-phosphatidylinositol 4,5-bisphosphate phosphodiesterase beta-1	calcium-binding protein(PC00060);guanyl-nucleotide exchange factor(PC00095);phospholipase(PC00022);signaling molecule(PC00113)	calcium ion binding(GO:0005488);guanyl-nucleotide exchange factor activity(GO:0005509);phospholipase activity(GO:0003824);receptor binding(GO:0030234);small GTPase regulator activity(GO:0005085)	NA	1
Q9NTG1	Polycystic kidney disease and receptor for egg jelly-related protein	G-protein modulator(PC00095);ion channel(PC00022);membrane-bound signaling molecule(PC00227)	Cation channel activity(GO:0005215)	NA	1
Q9P212	1-phosphatidylinositol 4,5-bisphosphate phosphodiesterase epsilon-1	calcium-binding protein(PC00060);guanyl-nucleotide exchange factor(PC00095);phospholipase(PC00022);signaling molecule(PC00113)	calcium ion binding(GO:0005488);guanyl-nucleotide exchange factor activity(GO:0005509);phospholipase activity(GO:0003824);receptor binding(GO:0030234);small GTPase regulator activity(GO:0005085)	NA	1
Q9P225	Dynein heavy chain 2, axonemal	hydrolase(PC00121);microtubule binding motor protein(PC00085)	microtubule motor activity(GO:0003824);structural constituent of cytoskeleton(GO:0016787)	intracellular(GO:0044464);microtubule(GO:0005622)	1
Q9P2N5	RNA-binding protein 27	RNA binding protein(PC00171)	RNA binding(GO:0005488)	NA	1
Q9UKA4	RNA-binding protein 27	kinase modulator(PC00095)	protein binding(GO:0005488)	cytoplasm(GO:0044464)	1

**Supplementary Table II:** List of proteins identified exclusively in the FF from OD group in comparison to PCOS group.

SwissProt ID	Name	Class	Molecular Function	Cellular compartment	Number of Patients
P43686	26S protease regulatory subunit 6B	hydrolase(PC00121)	protein binding(GO:0005488)	cytosol(GO:0044464);nucleus(GO:0005622);protein complex(GO:0005737)	2
Q8IWI9	MAX gene-associated protein	nucleic acid binding(PC00171);transcription factor(PC00218)	sequence-specific DNA binding transcription factor activity(GO:0005488)	NA	2
Q9Y4A5	Transformation/transcription domain-associated protein	non-receptor serine/threonine protein kinase(PC00220);nucleotide kinase(PC00137)	kinase activity(GO:0003824)	NA	2
A0AUZ9	KAT8 regulatory NSL complex subunit 1-like protein	NA	NA	NA	1
A5PLK6	Regulator of G-protein signaling protein-like	G-protein modulator(PC00095)	GTPase activity(GO:0003824);enzyme activator activity(GO:0016787);pyrophosphatase activity(GO:0003924)	cytoplasm(GO:0044464);plasma membrane(GO:0005622)	1
A6NJZ7	RIMS-binding protein 3C	NA	NA	NA	1
A6NNM3	RIMS-binding protein 3B	NA	NA	NA	1
O00268	Transcription initiation factor TFIID subunit 4	NA	NA	NA	1
O14641	Segment polarity protein dishevelled homolog DVL-2	enzyme modulator(PC00095);signaling molecule(PC00207)	receptor binding(GO:0005488)	cytosol(GO:0044464)	1
O14948	Transcription factor EC	basic helix-loop-helix transcription factor(PC00218)	NA	NA	1
O15294	UDP-N-acetylglucosamine--peptide N-acetylglucosaminyltransferase 110 kDa subunit	glycosyltransferase(PC00220)	transferase activity, transferring glycosyl groups(GO:0003824)	NA	1
O43187	Interleukin-1 receptor-associated kinase-like 2	NA	protein kinase activity(GO:0003824)	cytoplasm(GO:0044464);nucleus(GO:0005622)	1
O43451	Maltase-glucoamylase, intestinal	glucosidase(PC00121)	glucosidase activity(GO:0003824)	NA	1
O60229	Kalirin	guanyl-nucleotide exchange factor(PC00095);signaling molecule(PC00022)	guanyl-nucleotide exchange factor activity(GO:0003824);receptor binding(GO:0030234);small GTPase regulator activity(GO:0005085)	NA	1
O60318	Germinal-center associated nuclear protein	ATP synthase(PC00227);hydrolase(PC00068)	cation transmembrane transporter activity(GO:0005215);hydrogen ion transmembrane transporter activity(GO:0022857);hydrolase activity(GO:0008324)	NA	1
O60494	Cubilin	apolipoprotein(PC00219);cell adhesion molecule(PC00052);enzyme modulator(PC00069);extracellular matrix protein(PC00095);membrane-bound signaling molecule(PC00102);metalloprotease(PC00207);oxidase(PC00152);serine protease(PC00121);transporter(PC00190)	enzyme regulator activity(GO:0003824);lipid transporter activity(GO:0030234);metallopeptidase activity(GO:0005215);oxidoreductase activity(GO:0005319);receptor activity(GO:0016787);serine-type peptidase activity(GO:0008233);transmembrane transporter activity(GO:0008237)	extracellular matrix(GO:0031012);extracellular region(GO:0005576)	1
O75094	Slit homolog 3 protein	NA	NA	NA	1
O75901	Ras association domain-containing protein 9	membrane traffic protein(PC00150)	NA	NA	1
O94813	Slit homolog 2 protein	NA	NA	NA	1
O95206	Protocadherin-8	cadherin(PC00069)	calcium ion binding(GO:0005488)	NA	1
O95490	Latrophilin-2	G-protein coupled receptor(PC00197);antibacterial response protein(PC00021)	receptor activity(GO:0004872)	NA	1
O95613	Pericentrin	chromatin/chromatin-binding protein(PC00171);hydrolase(PC00009);kinase modulator(PC00077)	chromatin binding(GO:0005488);hydrolase activity(GO:0003682);kinase regulator activity(GO:0003824);nucleic acid binding(GO:0016787);protein binding(GO:0016740)	NA	1
O95661	GTP-binding protein Di-Ras3	small GTPase(PC00095)	GTPase activity(GO:0003824);protein binding(GO:0016787)	NA	1
O95835	Serine/threonine-protein kinase LATS1	annexin(PC00060);calmodulin(PC00050);non-receptor serine/threonine protein kinase(PC00131);transfer/carrier protein(PC00061)	protein kinase activity(GO:0003824)	intracellular(GO:0044464)	1
O95947	T-box transcription factor TBX6	nucleic acid binding(PC00171);transcription factor(PC00218)	sequence-specific DNA binding transcription factor activity(GO:0005488)	NA	1
P01857	Ig gamma-1 chain C region	NA	antigen binding(GO:0005488);receptor binding(GO:0003823)	extracellular space(GO:0005576);immunoglobulin complex(GO:0005615);plasma membrane(GO:0032991)	1
P06396	Gelsolin	non-motor actin binding protein(PC00085)	actin binding(GO:0005488);structural constituent of cytoskeleton(GO:0005515)	actin cytoskeleton(GO:0043226);intracellular(GO:0005856)	1
P07333	Macrophage colony-stimulating factor 1 receptor	NA	NA	NA	1
P0C7U3	Probable palmitoyltransferase ZDHHC11B	NA	NA	NA	1
P13761	HLA class II histocompatibility antigen, DRB1-7 beta chain	#N/D	#N/D	#N/D	1
P35527	Keratin, type I cytoskeletal 9	intermediate filament(PC00085);structural protein(PC00129)	structural constituent of cytoskeleton(GO:0005198)	intermediate filament cytoskeleton(GO:0043226);intracellular(GO:0005856)	1
P42345	Serine/threonine-protein kinase mT	non-receptor serine/threonine protein kinase(PC00220);nucleotide kinase(PC00137)	kinase activity(GO:0003824)	NA	1
P46013	Antigen KI-67	NA	NA	NA	1
P49750	YLP motif-containing protein 1	nucleic acid binding(PC00171)	nucleic acid binding(GO:0005488)	NA	1
P49792	E3 SUM	G-protein modulator(PC00095)	protein binding(GO:0005488);small GTPase regulator activity(GO:0005515)	NA	1
P51805	Plexin-A3	signaling molecule(PC00207);tyrosine protein kinase receptor(PC00197)	NA	NA	1
P98161	Polycystin-1	G-protein modulator(PC00095);ion channel(PC00022);membrane-bound signaling molecule(PC00227)	cation channel activity(GO:0005215)	NA	1
Q04637	Eukaryotic translation initiation factor 4 gamma 1	translation initiation factor(PC00171)	translation initiation factor activity(GO:0045182)	NA	1
Q08170	Serine/arginine-rich splicing factor 4	NA	NA	NA	1
Q08AE8	Protein spire homolog 1	actin family cytoskeletal protein(PC00085)	structural constituent of cytoskeleton(GO:0005198)	intracellular(GO:0044464)	1
Q13402	Unconventional myosin-VIIa	G-protein modulator(PC00095);actin binding motor protein(PC00022);cell junction protein(PC00085)	enzyme regulator activity(GO:0003824);motor activity(GO:0030234);protein binding(GO:0016787);structural constituent of cytoskeleton(GO:0016462)	actin cytoskeleton(GO:0043226);intracellular(GO:0005856);plasma membrane(GO:0015629)	1
Q13563	Polycystin-2	G-protein modulator(PC00095);ion channel(PC00022);membrane-bound signaling molecule(PC00227)	cation channel activity(GO:0005215)	NA	1
Q14643	Inositol 1,4,5-trisphosphate receptor type 1	ligand-gated ion channel(PC00227)	ligand-gated ion channel activity(GO:0005215);receptor activity(GO:0022857)	NA	1
Q14980	Nuclear mitotic apparatus protein 1	NA	NA	NA	1
Q15149	Plectin	non-motor actin binding protein(PC00085)	actin binding(GO:0005488);structural constituent of cytoskeleton(GO:0005515)	actin cytoskeleton(GO:0043226);intracellular(GO:0005856)	1
Q15413	Ryanodine receptor 3	ligand-gated ion channel(PC00227)	ligand-gated ion channel activity(GO:0005215);receptor activity(GO:0022857)	NA	1
Q15772	Striated muscle preferentially expressed protein kinase	G-protein coupled receptor(PC00197);immunoglobulin receptor superfamily(PC00021);immunoglobulin superfamily cell adhesion molecule(PC00090);protein phosphatase(PC00124)	NA	NA	1
Q16531	DNA damage-binding protein 1	damaged DNA-binding protein(PC00171);mRNA polyadenylation factor(PC00009)	damaged DNA binding(GO:0005488)	intracellular(GO:0044464);nucleus(GO:0005622);protein complex(GO:0043226)	1
Q2LD37	Uncharacterized protein KIAA1109	NA	NA	NA	1
Q3B7T1	Erythroid differentiation-related factor 1	NA	NA	NA	1
Q460N5	Poly [ADP-ribose] polymerase 14	nucleic acid binding(PC00171)	nucleic acid binding(GO:0005488)	NA	1
Q562E7	WD repeat-containing protein 81	esterase(PC00121);kinase inhibitor(PC00097);mRNA splicing factor(PC00095)	NA	NA	1
Q5JSL3	Dedicator of cytokinesis protein 11	guanyl-nucleotide exchange factor(PC00095)	guanyl-nucleotide exchange factor activity(GO:0003824);small GTPase regulator activity(GO:0030234)	NA	1
Q5T5C0	Syntaxin-binding protein 5	membrane trafficking regulatory protein(PC00150)	GTPase activity(GO:0003824);enzyme activator activity(GO:0016787);pyrophosphatase activity(GO:0003924)	cytoplasm(GO:0044464);plasma membrane(GO:0005622)	1
Q5TC82	Roquin-1	NA	NA	NA	1
Q5TZA2	Rootletin	kinase modulator(PC00095);viral protein(PC00140)	kinase regulator activity(GO:0003824);protein binding(GO:0016740)	NA	1
Q5UIP0	Telomere-associated protein RIF1	NA	NA	NA	1
Q5VST9	Obscurin	immunoglobulin receptor superfamily(PC00090);immunoglobulin superfamily cell adhesion molecule(PC00124);protein phosphatase(PC00069)	NA	NA	1
Q5VT52	Regulation of nuclear pre-mRNA domain-containing protein 2	kinase inhibitor(PC00095)	kinase inhibitor activity(GO:0003824);protein binding(GO:0030234)	NA	1
Q5VUA4	Zinc finger protein 318	NA	NA	NA	1
Q5VYK3	Proteasome-associated protein ECM29 homolog	kinase modulator(PC00095)	kinase regulator activity(GO:0003824);protein binding(GO:0016740)	NA	1
Q5VZM2	Ras-related GTP-binding protein B	small GTPase(PC00095)	GTPase activity(GO:0003824);pyrophosphatase activity(GO:0016787)	cytoplasm(GO:0044464);endosome(GO:0005622);lysosome(GO:0005737);membrane(GO:0043226);protein complex(GO:0005768);vacuole(GO:0005764)	1
Q66K64	DDB1- and CUL4-associated factor 15	NA	NA	NA	1
Q6P9F0	Coiled-coil domain-containing protein 62	#N/D	#N/D	#N/D	1
Q6ZN55	Zinc finger protein 574	KRAB box transcription factor(PC00218)	NA	NA	1
Q6ZT12	E3 ubiquitin-protein ligase UBR3	NA	NA	cytoplasm(GO:0044464);protein complex(GO:0005622)	1
Q6ZV29	Patatin-like phospholipase domain-containing protein 7	esterase(PC00121)	phospholipase activity(GO:0003824)	cytoplasm(GO:0044464);endoplasmic reticulum(GO:0005622)	1
Q70JA7	Chondroitin sulfate synthase 3	glycosyltransferase(PC00220)	transferase activity, transferring glycosyl groups(GO:0003824)	NA	1
Q7L523	Ras-related GTP-binding protein A	small GTPase(PC00095)	GTPase activity(GO:0003824);pyrophosphatase activity(GO:0016787)	cytoplasm(GO:0044464);endosome(GO:0005622);lysosome(GO:0005737);membrane(GO:0043226);protein complex(GO:0005768);vacuole(GO:0005764)	1
Q7Z494	Nephrocystin-3	NA	NA	microtubule(GO:0043226)	1
Q7Z794	Keratin, type II cytoskeletal 1b	intermediate filament(PC00085);structural protein(PC00129)	structural constituent of cytoskeleton(GO:0005198)	intermediate filament cytoskeleton(GO:0043226);intracellular(GO:0005856)	1
Q7Z7G8	Vacuolar protein sorting-associated protein 13B	NA	NA	NA	1
Q7Z7M0	Multiple epidermal growth factor-like domains protein 8	extracellular matrix linker protein(PC00102);receptor(PC00101);small GTPase(PC00197)	GTPase activity(GO:0003824);receptor activity(GO:0016787)	extracellular matrix(GO:0031012);extracellular region(GO:0005576)	1
Q86TS7	Putative UPF0730 protein encoded by LINC00643	NA	NA	NA	1
Q8IVE3	Pleckstrin homology domain-containing family H member 2	NA	NA	NA	1
Q8IYF3	Testis-expressed sequence 11 protein	chaperone(PC00072)	NA	NA	1
Q8IZD9	Dedicator of cytokinesis protein 3	guanyl-nucleotide exchange factor(PC00095)	guanyl-nucleotide exchange factor activity(GO:0003824);small GTPase regulator activity(GO:0030234)	NA	1
Q8N3S3	Putative homeodomain transcription factor 2	homeobox transcription factor(PC00218);nucleic acid binding(PC00116)	sequence-specific DNA binding transcription factor activity(GO:0005488)	NA	1
Q8N4F7	RING finger protein 175	ubiquitin-protein ligase(PC00142)	NA	Golgi apparatus(GO:0043226);cytoplasm(GO:0005794);endoplasmic reticulum(GO:0044464);nuclear outer membrane-endoplasmic reticulum membrane network(GO:0005622)	1
Q8N4S9	MARVEL domain-containing protein 2	tight junction(PC00070);transcription cofactor(PC00214)	sequence-specific DNA binding transcription factor activity(GO:0005488);transcription cofactor activity(GO:0003676)	plasma membrane(GO:0016020)	1
Q8N554	Zinc finger protein 276	NA	NA	NA	1
Q8N8Z8	Zinc finger protein 441	NA	NA	NA	1
Q8NA56	Tetratricopeptide repeat protein 29	guanyl-nucleotide exchange factor(PC00095);transmembrane receptor regulatory/adaptor protein(PC00022)	guanyl-nucleotide exchange factor activity(GO:0003824);small GTPase regulator activity(GO:0030234)	NA	1
Q8NE71	ATP-binding cassette sub-family F member 1	ATP-binding cassette (ABC) transporter(PC00227);hydrolase(PC00003);translation elongation factor(PC00121)	ATPase activity, coupled to transmembrane movement of substances(GO:0003824);translation elongation factor activity(GO:0016787);transmembrane transporter activity(GO:0042626)	NA	1
Q8NEZ4	Histone-lysine N-methyltransferase 2C	DNA binding protein(PC00171);methyltransferase(PC00009)	NA	NA	1
Q8NF91	Nesprin-1	non-motor actin binding protein(PC00085)	actin binding(GO:0005488);structural constituent of cytoskeleton(GO:0005515)	intracellular(GO:0044464)	1
Q8NFC6	Biorientation of chromosomes in cell division protein 1-like 1	NA	NA	NA	1
Q8NG31	Protein CASC5	NA	NA	NA	1
Q8NI35	InaD-like protein	NA	NA	NA	1
Q8TER5	Rho guanine nucleotide exchange factor 40	guanyl-nucleotide exchange factor(PC00095);signaling molecule(PC00022)	guanyl-nucleotide exchange factor activity(GO:0003824);receptor binding(GO:0030234);small GTPase regulator activity(GO:0005085)	NA	1
Q8WVC0	RNA polymerase-associated protein LE	DNA-directed RNA polymerase(PC00171)	NA	intracellular(GO:0044464);nucleus(GO:0005622)	1
Q8WWG9	Potassium voltage-gated channel subfamily E member 4	NA	NA	NA	1
Q92823	Neuronal cell adhesion molecule	G-protein coupled receptor(PC00197);immunoglobulin receptor superfamily(PC00021);immunoglobulin superfamily cell adhesion molecule(PC00090);protein phosphatase(PC00124)	NA	NA	1
Q969F9	Hermansky-Pudlak syndrome 3 protein	NA	NA	NA	1
Q969V4	Tektin-1	NA	NA	NA	1
Q96B21	Transmembrane protein 45B	NA	NA	NA	1
Q96ID5	Immunoglobulin superfamily member 21	immunoglobulin receptor superfamily(PC00090);immunoglobulin superfamily cell adhesion molecule(PC00124);protein phosphatase(PC00069)	NA	NA	1
Q96MB7	Putative nuclease HARBI1	NA	NA	NA	1
Q96NH3	Protein broad-minded	NA	NA	NA	1
Q96PX9	Pleckstrin homology domain-containing family G member 4B	guanyl-nucleotide exchange factor(PC00095);signaling molecule(PC00022)	guanyl-nucleotide exchange factor activity(GO:0003824);receptor binding(GO:0030234);small GTPase regulator activity(GO:0005085)	NA	1
Q9BQ52	Zinc phosphodiesterase ELAC protein 2	NA	endoribonuclease activity(GO:0003824)	NA	1
Q9BSC4	Nucleolar protein 10	NA	NA	NA	1
Q9GIY3	HLA class II histocompatibility antigen, DRB1-14 beta chain	#N/D	#N/D	#N/D	1
Q9GZQ4	Neuromedin-U receptor 2	G-protein coupled receptor(PC00197)	receptor activity(GO:0004872)	NA	1
Q9H0X9	Oxysterol-binding protein-related protein 5	NA	NA	NA	1
Q9H1A4	Anaphase-promoting complex subunit 1	ligase(PC00142)	ligase activity(GO:0003824)	NA	1
Q9H254	Spectrin beta chain, non-erythrocytic 4	non-motor actin binding protein(PC00085)	actin binding(GO:0005488);structural constituent of cytoskeleton(GO:0005515)	intracellular(GO:0044464)	1
Q9H7M6	Zinc finger SWIM domain-containing protein 4	NA	NA	NA	1
Q9NRL2	Bromodomain adjacent to zinc finger domain protein 1A	acetyltransferase(PC00220);chromatin/chromatin-binding protein(PC00038)	acetyltransferase activity(GO:0003824);chromatin binding(GO:0016740);nucleic acid binding(GO:0016746)	NA	1
Q9NZC2	Bromodomain adjacent to zinc finger domain protein 1A	immunoglobulin receptor superfamily(PC00090)	receptor activity(GO:0004872)	NA	1
Q9P107	GEM-interacting protein	NA	NA	NA	1
Q9P227	Rho GTPase-activating protein 23	NA	GTPase activity(GO:0003824);enzyme activator activity(GO:0016787);pyrophosphatase activity(GO:0003924)	NA	1
Q9UFD9	RIMS-binding protein 3A	NA	NA	NA	1
Q9UFH2	Dynein heavy chain 17, axonemal	hydrolase(PC00121);microtubule binding motor protein(PC00085)	microtubule motor activity(GO:0003824);structural constituent of cytoskeleton(GO:0016787)	intracellular(GO:0044464);microtubule(GO:0005622)	1
Q9UGM6	Tryptophan--tRNA ligase, mitochondrial	NA	NA	cytoplasm(GO:0044464)	1
Q9UHT4	Putative uncharacterized protein PR	#N/D	#N/D	#N/D	1
Q9UKN7	Unconventional myosin-XV	G-protein modulator(PC00095);actin binding motor protein(PC00022);cell junction protein(PC00085)	enzyme regulator activity(GO:0003824);motor activity(GO:0030234);protein binding(GO:0016787);structural constituent of cytoskeleton(GO:0016462)	actin cytoskeleton(GO:0043226);intracellular(GO:0005856);plasma membrane(GO:0015629)	1
Q9ULL8	Protein Shroom4	ligand-gated ion channel(PC00227)	ligand-gated ion channel activity(GO:0005215);receptor activity(GO:0022857)	NA	1
Q9Y2H0	Disks large-associated protein 4	transmembrane receptor regulatory/adaptor protein(PC00226)	NA	NA	1
Q9Y493	Zonadhesin	cell adhesion molecule(PC00069);extracellular matrix glycoprotein(PC00102)	NA	extracellular matrix(GO:0031012);extracellular region(GO:0005576)	1
Q9Y5T5	Ubiquitin carboxyl-terminal hydrolase 16	#N/D	#N/D	#N/D	1
Q9Y5Y6	Suppressor of tumorigenicity 14 protein	annexin(PC00060);calmodulin(PC00050);peptide hormone(PC00131);protease inhibitor(PC00061);receptor(PC00207);serine protease(PC00179)	NA	NA	1
Q9Y6U3	Adseverin	non-motor actin binding protein(PC00085)	actin binding(GO:0005488);structural constituent of cytoskeleton(GO:0005515)	actin cytoskeleton(GO:0043226);intracellular(GO:0005856)	1

**Supplementary Table III:** Canonical Pathways matched for the unique and increased proteins of the FF from PCOS and OD groups.

	PCOS Group			OD Group		
Canonical Pathways	p-value	Overlap		*p*-value	Overlap	
LXR/RXR Activation	9,04E^-11^	7.0%	9/128			
FXR/RXR Activation	1,67E^-10^	6.6%	9/137			
Extrinsic Prothrombin Activation Pathway	1,70E^-07^	22.2%	4/18			
Acute Phase Response Signaling	5,21E^-07^	4.1%	7/171	3,06E^-03^	2.9%	5/171
Intrinsic Prothrombin Activation Pathway	1,48E^-06^	13.3%	4/30			
Allograft Rejection Signaling				1,29E^-02^	3.5%	3/85
Communication between Innate and Adaptive Immune Cells				1,50E^-02^	3.3%	3/90
Crosstalk between Dendritic Cells and Natural Killer Cells				1,50E^-02^	3.3%	3/90
Germ Cell-Sertoli Cell Junction Signaling				1,86E^-02^	2.3%	4/176
